# Perceived Life Meaning and Purpose and Its Association with Mental and Physical Health among Family Caregivers

**DOI:** 10.1093/geroni/igab046.2940

**Published:** 2021-12-17

**Authors:** Yu-Ping Chang, Loralee Sessanna, Young Sik Seo

**Affiliations:** 1 University at Buffalo, Buffalo, New York, United States; 2 Roswell Park Comprehensive Cancer Center, Buffalo, New York, United States

## Abstract

Evidence suggests that having a sense of life meaning and purpose is related to physical health. However, the association between life meaning and purpose and physical and mental health among family caregivers remains unclear. This study aimed to examine whether family caregivers’ perceived life meaning and purpose was associated with their physical and mental health (depression and anxiety). The National Study of Caregiving (NSOC) III cross-sectional survey (2017, N = 2,652) was utilized. One item was used to measure family caregivers’ perceived life meaning and purpose and two composite variables were generated to measure depression and anxiety. Physical health was assessed by questions including pain, breathing problems, limited arm/leg strength, low energy, and sleep problems. Weighted logistic regression analyses with covariate adjustments (i.e., caregiver’s age, sex, and race/ethnicity) were conducted to examine the association among family caregivers’ perceived life meaning and purpose, mental and physical health. Results indicated that family caregivers’ perceived life meaning and purpose was associated with a lower probability of having depressive symptoms (OR, .29, 95% Confidence Interval [CI], .15, .57) and anxiety (OR, .43, 95% CI, .23, .79). Furthermore, perceived life meaning and purpose was associated with a lower probability of having breathing problems (OR, .50, 95% CI [.25, .99]). Findings suggest that having a strong sense of life meaning and purpose is linked to better mental health and physical symptoms. Further research is needed to determine the mechanism regarding how life meaning and purpose may improve mental and physical health among family caregivers.

